# Irritable Heart

**Published:** 1919

**Authors:** 


***Irritable Heart.*** By L. M. WARFIELD and F. M. SMITH. A Preliminary Report of Studies on the Irritable Heart. *Journ. of the American Medical Association*, Nov. 30, 1918.

Careful studies of reports of Lewis and his associates lead the authors to believe that they were not dealing with the same class of patients. Lewis' patients were invalided from the front where over-exertion and shell shock were factors. Their own patients came from civil life, where they had worked under no great strain. Lewis was able to return 20 per cent. to active duty. Their experience coincides with that of Major Robey, who found that his patients were not benefited by graded exercises. The patients had been doing steady work without strain or sudden call on the heart, and some of them reported that they had found certain work too hard for them, and had voluntarily sought easier and, at times, sedentary jobs. This fact they consider important. The authors have studied 435 cases, but 200 of these were referred for examination elsewhere, and the remaining 235 form the basis of this report. A careful history from the man who has tachycardia is almost the most important factor in the question of his fitness for the Army. Those men who have shortness of breath, feel exhaustion out of proportion to the effort made, palpitation of the heart, precordial pain and dizziness in civil life, have always failed in their military training. The authors' patients, almost without exception, complained of subjective symptoms leading back into their previous life, sometimes as far as to childhood. Patients with frank exophthalmic goiter, hyperthyroidism, and patent pulmonary tuberculosis were rejected at once, and the cases here reported on were those who had tachycardia but could not be at once rejected. One hundred and five were put in the hospital and given graded exercises, of whom sixty-five were discharged as unfit, and eighteen only accepted for military services. The irritable heart is frequently a symptom of well recognized disease. The diagnosis of pulmonary tuberculosis was made in eighty-eight; seventy-three patients were discharged with a diagnosis of hyperthyroidism, all with the definite eye signs, enlarged thyroid gland, and occasionally exophthalmus. There was tremor of the hand and tachycardia, and usually increased systolic pressure. Thirty cases gave a typical history of effort syndrome with nothing found to account for it. Twenty-one were discharged from the examining barracks, and the authors think that possibly some would have been found tuberculous by longer observation. Others suffered from various morbid conditions, and twenty-one are still under observation.

The graded exercises are valuable in determining a man's fitness and establishing the diagnosis. Those men who are unable to do the simplest exercises without distress, and do not improve, are obviously unfit. Those who have made complaints of subjective symptoms in civil life have all complained of the graded exercises, and when tuberculosis is the cause the response is poorest. At present, the authors feel safe in saying that it is possible to detect the irritable heart prior to full acceptance for military service; that is, by careful history taking, giving special attention to cardiac symptoms and pounding on slight exertions, and also by noticing the unusual physical response to the hopping exercises and disproportionate increase of pulse rate from test.

